# Cyclist route choice, traffic-related air pollution, and lung function: a scripted exposure study

**DOI:** 10.1186/1476-069X-12-14

**Published:** 2013-02-07

**Authors:** Sarah Jarjour, Michael Jerrett, Dane Westerdahl, Audrey de Nazelle, Cooper Hanning, Laura Daly, Jonah Lipsitt, John Balmes

**Affiliations:** 1 University of California, Berkeley, 50 University Hall, Berkeley, CA, 94720, USA; 2 Cornell University, Ithaca, NY, USA; 3 Centre for Research in Environmental Epidemiology (CREAL), Doctor Aiguader, 88, Barcelona, Spain; 4 University of California, San Francisco, 1001 Potrero Ave, SFGH 30, San Francisco, CA, 94110, USA

**Keywords:** Bicycle boulevards, Active transportation, Air pollution, Lung function

## Abstract

**Background:**

A travel mode shift to active transportation such as bicycling would help reduce traffic volume and related air pollution emissions as well as promote increased physical activity level. Cyclists, however, are at risk for exposure to vehicle-related air pollutants due to their proximity to vehicle traffic and elevated respiratory rates. To promote safe bicycle commuting, the City of Berkeley, California, has designated a network of residential streets as “Bicycle Boulevards.” We hypothesized that cyclist exposure to air pollution would be lower on these Bicycle Boulevards when compared to busier roads and this elevated exposure may result in reduced lung function.

**Methods:**

We recruited 15 healthy adults to cycle on two routes – a low-traffic Bicycle Boulevard route and a high-traffic route. Each participant cycled on the low-traffic route once and the high-traffic route once. We mounted pollutant monitors and a global positioning system (GPS) on the bicycles. The monitors were all synced to GPS time so pollutant measurements could be spatially plotted. We measured lung function using spirometry before and after each bike ride.

**Results:**

We found that fine and ultrafine particulate matter, carbon monoxide, and black carbon were all elevated on the high-traffic route compared to the low-traffic route. There were no corresponding changes in the lung function of healthy non-asthmatic study subjects. We also found that wind-speed affected pollution concentrations.

**Conclusions:**

These results suggest that by selecting low-traffic Bicycle Boulevards instead of heavily trafficked roads, cyclists can reduce their exposure to vehicle-related air pollution. The lung function results indicate that elevated pollutant exposure may not have acute negative effects on healthy cyclists, but further research is necessary to determine long-term effects on a more diverse population. This study and broader field of research have the potential to encourage policy-makers and city planners to expand infrastructure to promote safe and healthy bicycle commuting.

## Background

A shift from motor vehicle use to active transportation such as bicycling would help reduce traffic volume and related air pollution emissions. Short trips (under three miles) in particular have been identified as a good target for this travel mode shift; reducing these vehicle miles traveled in the United States by 0.8-1.8% would save an estimated 20,000-46,000 tons/day of CO_2_ equivalent of exhaust emissions nation-wide (a 0.80-1.78 percent reduction) [1]. Such a shift may improve public health through increased physical activity, as bicycle commuting is also inversely correlated with overweight and obesity [2]. Despite its potential benefits, bicycling remains an underutilized method of transportation. In the United States, cycling accounts for less than 1% of trips [3].

The environmental and public health benefits of bicycle commuting must be weighed against the associated risks such as traffic accidents and air pollution exposure for the cyclist. Vehicle traffic is associated with the emission of multiple air pollutants and related health effects [4,5]. While measured concentrations of PM_2.5_, elemental carbon, and ultrafine particulate matter are similar or higher inside vehicles than on bicycles [6,7], cyclists’ minute ventilation has been recorded at two to four times that of car passengers, leading to overall higher inhaled doses of pollutants for trips of the same length [6,8].

Few studies have examined whether these elevated exposures relate to adverse health effects. A study in the Netherlands that evaluated the relationships between vehicle exhaust pollutant exposures during bicycle commuting and respiratory health effects yielded inconclusive results [9]. In contrast, a Canadian study of 42 bicyclists found an inverse relationship between heart rate variability (standard deviation of normal-to-normal interval) and pollutant exposure (NO_2_ and O_3_ concentrations) as well as an association between ultrafine particulate matter (UFPM) and decreased high-frequency power [10]. Decreased heart rate variability is associated with morbidity and mortality from cardiopulmonary disease [11], indicating that pollutant exposure associated with bicycling may have an adverse effect on cardiovascular health.

Our study builds on previous work by comparing the pollutant exposures and associated lung function for cyclists on high-traffic and low-traffic routes in the City of Berkeley, California. Routes were chosen to compare exposures on normal major roads to those on bicycle boulevards. Berkeley’s bicycle boulevard system is a network of low-volume residential streets designated as “bicycle priority routes.” To our knowledge, this is the first study to look at the difference between a study route chosen to follow only cyclist-designated streets and a study route on regular busy streets. This is significant because bicycle boulevards can be designated without the large capital investments often associated with bicycle infrastructure and may therefore provide a low cost means of promoting increased bicycling. Our study aims to demonstrate that there is a potential health benefit associated with choosing bicycle boulevards instead of arterial streets. We hypothesized that cyclists would be exposed to higher concentrations of particulate matter and other vehicle exhaust pollutants on high-traffic routes as compared to cyclists on low-traffic routes and that this elevated exposure may result in reduced lung function.

## Methods

### Study site

**Figure 1:**
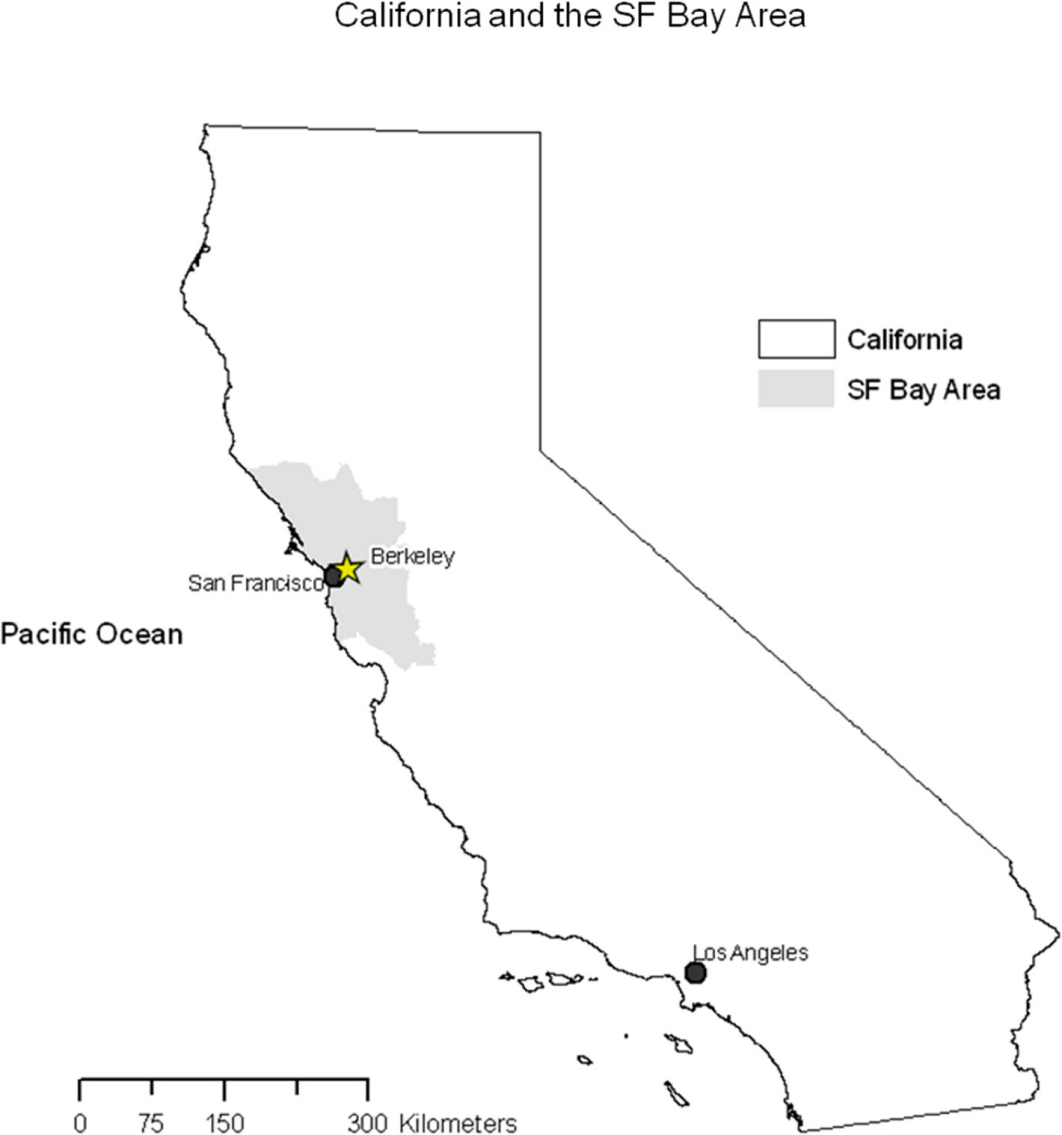
The City of Berkeley is in the San Francisco Bay Area in Northern California.

We conducted this study in the City of Berkeley (population ~112,580). Berkeley is within the San Francisco Metropolitan Area (population ~7.3 million) in Northern California (see Figure 1).

The City has a temperate Mediterranean climate, but regularly experiences strong westerly winds from the Pacific Ocean. As a result, the City has relatively low background pollution, usually under the National Ambient Air Quality Standards adopted by the U.S. Environmental Protection Agency (EPA) [12] for ozone, carbon monoxide, sulfur dioxide, particulate matter (10 μm diameter or PM_10_), and fine particulate matter (2.5 μm diameter or PM_2.5_) [13]. In 2000, the City implemented the Berkeley Bicycle Plan, which established a network of seven bicycle boulevards: low-volume residential streets with signs, pavement markings, and traffic calming devices designed to promote safe and convenient bicycle commuting and walking [14].

Low background air pollution, combined with an explicit network of bicycle boulevards, makes Berkeley a good location to conduct bicycle exposure studies. The University of California, Berkeley, generates approximately 30,000 trips through the downtown area per weekday, 52% of which are individuals driving alone and 11% of which are carpools (the remaining 37% are divided between public transportation, walking, and cycling) [15].

### Routes

**Figure 2:**
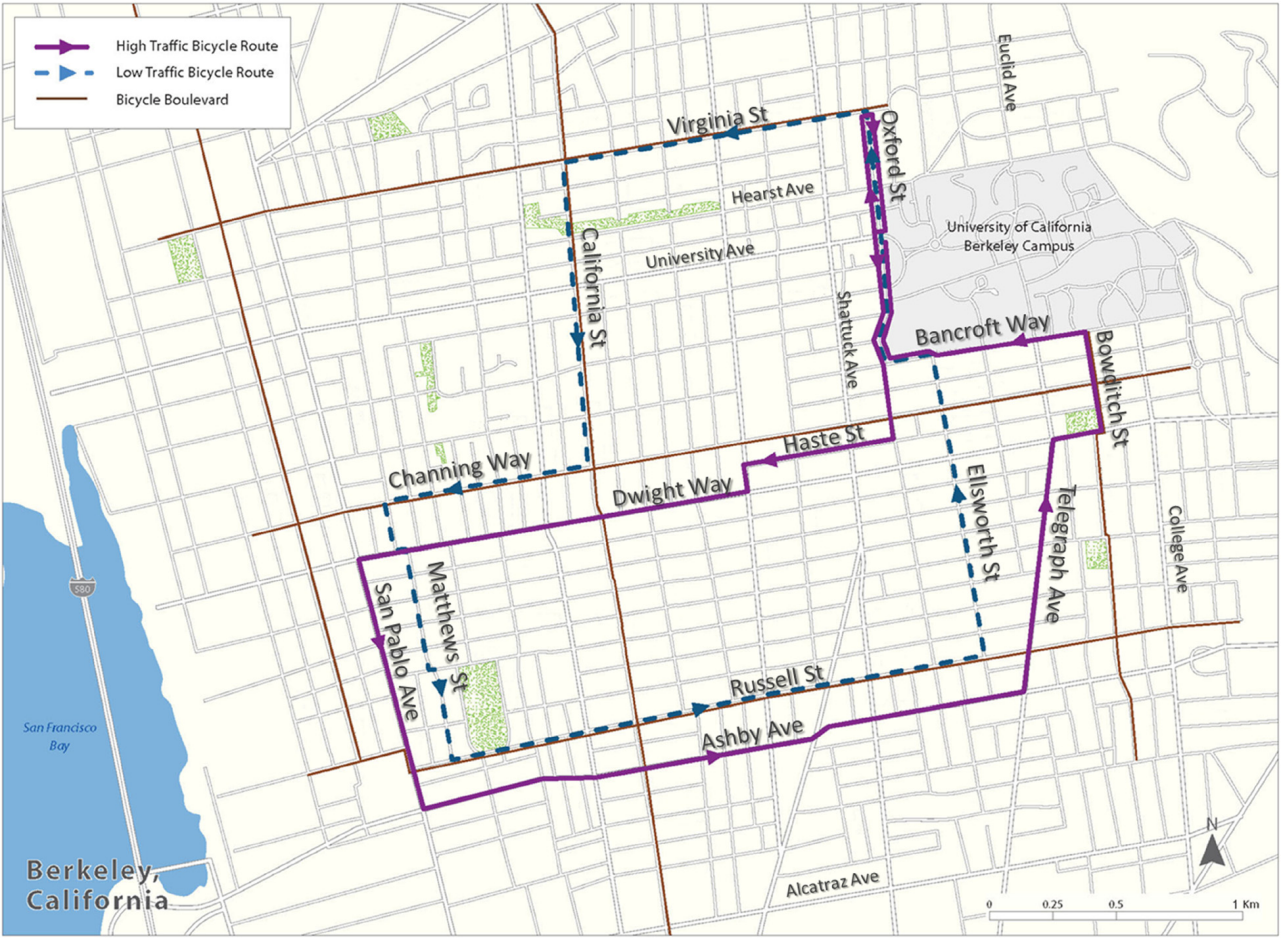
**Low and high-traffic routes in Berkeley, California.** Both routes start and end at University Hall on the west side of the UC Berkeley campus.

Data were collected on weekdays in April-June 2011 during morning commute hours (8:00–10:00 AM). On each study day, a pair of participants bicycled together on either a low-traffic or high-traffic study route. The two routes were similar in length (8–9.5 km) and elevation change (~61 m). The high-traffic route followed busy streets with more truck and bus traffic, while the low-traffic route followed residential streets, all designated by the City of Berkeley as bicycle boulevards (Figure 2). Traffic counts on the high-traffic route range from ~10,000 vehicles per day (vpd) on Dwight Way to ~26,000 vpd on San Pablo Ave [16]. Traffic counts for the low-traffic route were not available, but the Berkeley Bicycle Plan deemed streets with low traffic volumes, defined as less than 3,000-4,000 vehicles per day, as appropriate for bicycle boulevards. In many parts of the low-traffic route, counts are likely to be less than 1,500 vpd [14].

We selected these routes to compare low and high traffic exposures, so they do not necessarily model realistic commuting scenarios. An actual commute would likely follow a combination of residential and busy streets, depending on the origin and destination of the cyclist and personal preferences. Although differences in cyclist pace and riding habits could not be completely controlled, participants were asked to cycle at a normal commuting pace and observe bicycle traffic rules (e.g., stopping at stop signs and signaling turns).

### Participants

We recruited 15 adults (age 23–48) by word of mouth and departmental email lists from the UC Berkeley School of Public Health. Subjects completed a preliminary screening survey prior to study enrollment. Exclusion criteria included respiratory (including asthma), cardiovascular, or other chronic conditions, and smoking (current or recent). We only enrolled individuals who were already regular cyclists in the City of Berkeley (defined as cycling more than once a week). These selection criteria were used to minimize risk of injury due to unfamiliarity with Berkeley streets or cycling inexperience, or adverse acute health effects. Participants were asked to refrain from alcohol and caffeine consumption starting the evening before they were scheduled to ride and were asked to avoid biking to the study site to minimize potential pre-study cycling exposure. Participants primarily walked or drove to the study site, with commute times of 10–20 minutes. The UC Berkeley Center for Protection of Human Subjects approved this study, and all participants provided written informed consent.

### Exposure measurements

We used a condensation particle counter (CPC) (TSI Model 3007, Shoreview, MN) to measure ultrafine particulate matter (UFPM) (0.01 to 1.0 μm diameter) with a logging interval of 10 seconds. Carbon monoxide (CO) was recorded using a Q-Trak (TSI Model VelociCalc/Q-Trak 7565, Shoreview, MN), also with a logging interval of 10 seconds. A DustTrak (TSI Model 8520, Shoreview, MN) fitted with a PM_2.5_ inlet was used to measure fine particulate matter (PM_2.5_) (less than 2.5 μm diameter) with a logging interval of 10 seconds, and a microaethelometer (Magee Scientific Model AE-51, Berkeley, CA) with a logging interval of 1 second was used to measure black carbon (BC).

These devices were placed in a rear basket (40.5 × 33.5 × 24.1 cm) of a bike. Together they weighed approximately 9.5 kg (21 pounds). One subject carried a GPS (GPSMAP 60CSx, Garmin, Olathe, KS) to track location. All monitoring devices were synced to GPS time before each bike ride. Data were collected on each machine’s internal memory. After each bike ride, data were downloaded onto a computer using the manufacturers’ software.

### Meteorology

We downloaded meteorological information from the downtown Berkeley weather station (weather.berkeley.edu) for each study day. We calculated the median of the wind speeds for all 19 study days. Days with wind speeds above the median (6.83 mph = 3.05 m/s) were classified as high-wind; days with wind speeds below the median were classified as low-wind. The Q-Trak monitor also recorded temperature and humidity.

### Data processing

We adjusted PM_2.5_ measurements for humidity (RH, as measured by the Q-Trak monitor) using the correction factor (CF) [17]:

$$\;\mathrm{CF}=1+0.25\frac{RH^{2}}{\left(1-\mathrm{RH}\right)}$$

Adjusted PM_2.5_ = Uncorrected PM_2.5_/CF.

We also calibrated PM_2.5_ measurements to account for the lower precision of the DustTrak (as compared to US EPA designated Federal Reference Method measurements) using the equation developed in Yanosky, et al. (2002) [18]:

y=0.33x+2.25

Where x equals recorded values and y equals corrected values.

One-second BC data were processed to remove erroneous spikes that occurred when the microaethelometer was exposed to sudden movement or vibration [19]. The processed data were smoothed to a 29-second moving average, and every tenth point was matched with the other measurements taken in 10-second intervals. After data processing, the few remaining negative BC and PM_2.5_ values were assumed to be noise, again resulting from equipment error, and set as missing measurements. BC measurements below the minimum detection limit (0.1 μg) were also removed.

As described in a previous vehicular emissions study, the CPC (3007 model) undercounts UFPM when concentrations are above 100,000 particles/cm^3^. We used the correction equation:

$$\;y=38456e^{0.00001}$$

Where x equals recorded values (over 100,000) and y equals corrected values [20].

### Exposure mapping

To map the spatial variation along each route, we aggregated data points by 50 meter segments. The points were added to a Berkeley base map (WGS_1984_UTM_Zone_10N) in ArcMap 10 (ArcGIS 10, Esri, Redlands, CA). The bike route lines were buffered by 50 meters and divided into 50 m × 50 m block segments (high-traffic route 51.54 m × 50 m; low-traffic route 52.76 m × 50 m), then the data points were assigned to block segments by spatial join and aggregated using the Dissolve tool, which averaged the pollutant measurements of all the points within a block segment. The low-traffic route was broken down into 170 segments, and the high-traffic route was 200 segments long. We calculated a moving average of approximately 150 meters for each block segment (i.e., a centered moving average of three 50 m block segments). These averages were only used for qualitative purposes.

### Statistical analysis

Exposure differences and percent differences between high-traffic and low-traffic rides were calculated for each subject. We compared the average high-traffic to low-traffic exposures by subject using a pairwise *t*-test and excluding subjects who were missing pollutant measurements from one or both rides due to equipment malfunctions. We used Stata (Version 10, StataCorp, College Station, TX) for all statistical analyses.

### Health outcome measurements

Each subject completed the high-traffic route and the low-traffic route on separate days. Three spirometry sessions per ride were recorded. Specifically, lung function was evaluated before, immediately after, and about 4 hours after each bike ride using an EasyOne Spirometer. Trained research personnel coached each study subject through at least three (up to eight) rounds of forceful inhalation and exhalation following the performance criteria recommended by the American Thoracic Society (ATS) and European Respiratory Society (ERS) [21].

The spirometry data were reviewed by an experienced pulmonologist. Six spirometry sessions did not meet ATS/ERS standards for reproducibility and were omitted from analysis. One of these sessions was a pre-ride baseline measurement, so the corresponding post-ride and 4-hour follow-up measurements were also omitted.

Differences between baseline, post-ride, and 4-hour follow-up measurements were calculated from the quality-assured dataset. We compared changes in lung function after the high-traffic and low-traffic routes using a pairwise *t*-test (by subject).

## Results

### Participants and route completion

Of the 15 participants, four were female and 11 were male (Table 1). All participants completed both routes. The mean age was 32 years, and compliant with the exclusion criteria none of the participants reported respiratory health conditions, cardiovascular conditions, or recent or current smoking habits. The low-traffic route was completed nine times and the high-traffic route was completed 10 times over 19 separate study days (April 14 – June 23, 2011). On the low-traffic route, the average travel time was 37.6 minutes (range 31.5 – 44.2 minutes). On the high-traffic route, the average travel time was 40.2 minutes (range 28.7 – 49.5 minutes).

**Table 1:** Participant demographics and baseline lung function

**Characteristic**	
Female – n (%)	4 (26.7%)
Age – years	
Mean ± SD	32.2 ± 6.67
Range	23 – 48
Height – meters	1.75 ± 0.09
BMI – kg/m^2^	22.03 ± 1.92
Predicted FVC – liters	4.70 ± 0.84
Baseline FVC – liters	
Mean ± SD	4.95 ± 0.78
Range	3.45 – 6.75
Predicted FEV_1_ – liters	4.00 ± 0.74
Baseline FEV_1_ – liters	
Mean ± SD	3.94 ± 0.64
Range	2.01 – 5.13

*N = 15 participants.

### Air pollutant exposure

Black carbon measurements were recorded on all study days. Ultrafine particulate matter and carbon monoxide measurements were obtained for all but one day. Due to equipment failure, fine particulate matter measurements were missing for five study days. Two days of measurement were excluded due to rain and a flat tire.

For all pollutants, averages were higher on the high traffic route (Table 2). In a subject-by-subject comparison of high-traffic and low-traffic measurements (Table 3), almost all subjects experienced higher pollution levels on the high-traffic route. For UFPM and BC, three participants had lower average exposure on the high-traffic route. For CO two participants had lower average exposure on the high-traffic route. Percent differences between high and low-traffic exposures varied greatly.

**Table 2:** **Average, standard deviation, range, and 5-95**^**th**^**percentile of pollutant exposures for low and high-traffic routes**

**Pollutant**	N*	**Mean ± SD**	**Min**	**Max**	**5**^**th**^**– 95**^**th**^
UFPM – #/cm^3^					
Low-traffic	9	14,311 ± 15,381	2,771	376,495	4,621-29,882
High-traffic	9	18,545 ± 42,482	1,900	1,033,188	4,148-51,265
CO – ppm					
Low-traffic	8	0.79 ± 0.39	0.20	4.90	0.40-1.50
High-traffic	10	0.90 ± 0.64	0.10	10.60	0.40-1.90
PM_2.5_ – μg/m^3^					
Low-traffic	6	4.88 ± 1.41	2.25	20.96	2.65-6.84
High-traffic	8	5.12 ± 1.86	2.25	27.40	2.94-7.10
BC – μg/m^3^					
Low-traffic	9	1.76 ± 2.58	0.11	63.83	0.50-4.03
High-traffic	10	2.06 ± 3.23	0.1	53.53	0.37-5.06

* N = number of sampling days with valid pollutant data (varied due to equipment failures).

**Table 3:** Average high and low-traffic air pollution exposures by subject

	**UFPM (#/cm**^**3**^**)**			**PM**_**2.5**_**(μg/m**^**3**^**)**			**CO (ppm)**			**BC(μg/m**^**3**^**)**		
**Sub-ject**	**High-traffic average**	**Difference (high-low)**	% Diff. (Diff/Avg)*100	**High-traffic average**	**Difference (high-low)**	% Diff. (diff/avg)*100	**High-traffic average**	**Difference (high-low)**	% Diff. (diff/avg)*100	**High-traffic average**	**Difference (high-low)**	% Diff. (diff/avg)*100
	**Low-traffic average**			**Low-traffic average**			**Low-traffic average**			**Low-traffic average**		
1	8992.69	1554.56	18.9	3.69	−0.75	−18.5	0.79	0.13	18.5	1.59	0.39	27.8
	7438.13			4.43			0.66			1.20		
2	15163.01	−2836.17	−17.1	5.46	0.29	5.5	0.88	0.15	19.1	3.57	1.61	58.1
	17999.18			5.17			0.73			1.96		
3	23154.37	9140.15	49.2	3.24			1.19	0.43	43.6	1.77	0.16	9.6
	14014.22						0.76			1.61		
4	23154.37	9762.57	53.4	3.24	−2.69	−58.7	1.19	0.42	43.4	1.77	0.00	.18
	13391.8			5.93			0.77			1.77		
5	19443.05	12004.91	89.3				0.92	0.27	33.9	2.78	1.58	79.2
	7438.13			4.43			0.66			1.20		
6				4.32	1.07	28.2	0.79			1.99	0.35	19.5
	14141.43			3.25						1.64		
7	19443.05	6443.09	39.7				0.92	0.21	25.3	2.78	1.57	78.9
	12999.95			4.89			0.72			1.21		
8	13268.25	−745.98	−5.5				0.92	0.16	19.0	1.79	0.18	10.7
	14014.22						0.76			1.61		
9	15234.06	−2643.94	−16.0	6.07			0.94	−0.18	−18.0	1.69	−0.78	−37.5
	17878.00						1.12			2.47		
10	35506.35	22834.39	94.8	6.01			0.94	0.22	26.6	2.48	0.50	22.7
	12671.96						0.72			1.97		
11	35506.35	20343.34	84.5	6.01			0.94	0.22	26.6	2.48	0.50	22.7
	12671.96						0.72			1.97		
12	15234.06	1092.63	7.4	6.07	2.82	60.6	0.94			1.69	0.05	3.0
	14141.43			3.25						1.64		
13	18728.86	3134.31	18.3	5.89	0.34	5.9	0.72	−0.08	−11.1	1.99	0.05	2.5
	15594.55			5.55			0.81			1.94		
14	18203.17	2608.62	15.4	3.79	−1.76	−37.7	0.85	0.04	5.0	1.56	−0.38	−21.9
	15594.55			5.55			0.81			1.94		
15	18203.17	4811.37	30.5	3.79	−2.14	−44.0	0.85	0.08	10.3	1.56	−0.21	−12.6
	13391.80			5.93			0.77			1.77		

The results of the pairwise (by subject) *t*-test are shown in Table 4. For ultrafine particulate matter, black carbon, and carbon monoxide, high-traffic averages were higher than low-traffic. For PM_2.5_, the high-traffic average was lower than the low-traffic average, but this difference was not statistically significant, as we would expect from a pollutant with lower variability at small spatial scales.

**Table 4:** **Paired *t*-test by subject. Average pollutant exposure for each subject’s high-traffic ride vs. low-traffic ride average**

	N*	**Mean**		**Standard error of the mean**		**p-value**	**95**% **CI of difference**	
		**Low-traffic**	**High-traffic**	**Low-traffic**	**High-traffic**		**Lower**	**Upper**
UFPM (#/cm^3^)	14	13517.13	19945.34	826.28	2022.87	0.01	1656.84	11199.58
PM_2.5_ (μg/m^3^)	8	4.88	4.53	0.40	0.39	0.60	−1.90	1.19
BC (μg/m^3^)	15	1.73	2.10	0.90	0.15	0.06	−0.02	0.77
CO (ppm)	13	0.77	0.93	0.03	0.04	0.006	0.05	0.26

*N = number of subjects with high & low measurements for pollutant (i.e., number of pairs) (varied due to equipment failures).

### Exposure mapping

**Figure 3:**
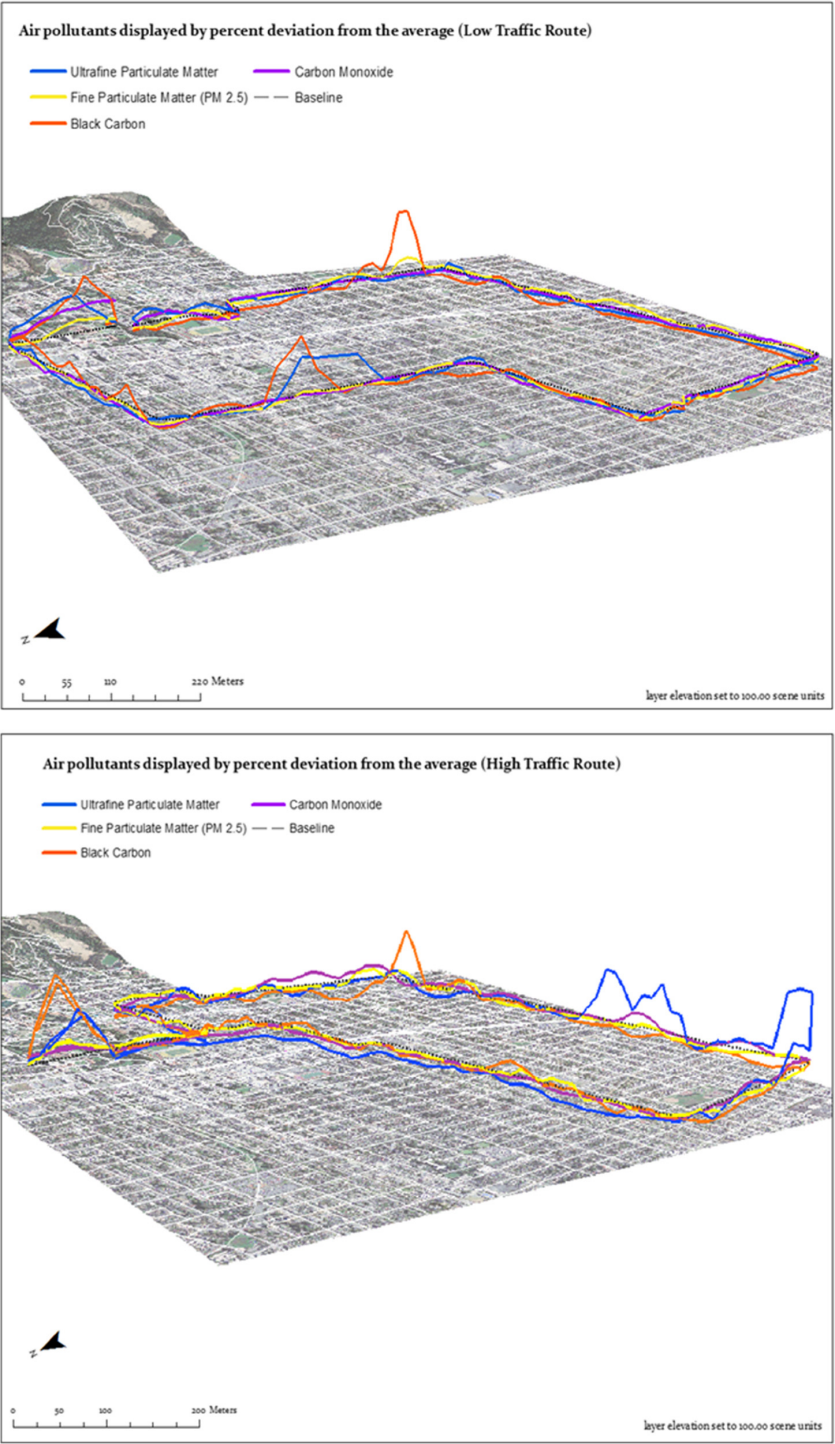
Pollutant concentrations (percent deviation from average, or “baseline”) on the low and high-traffic routes.

**Figure 4:**
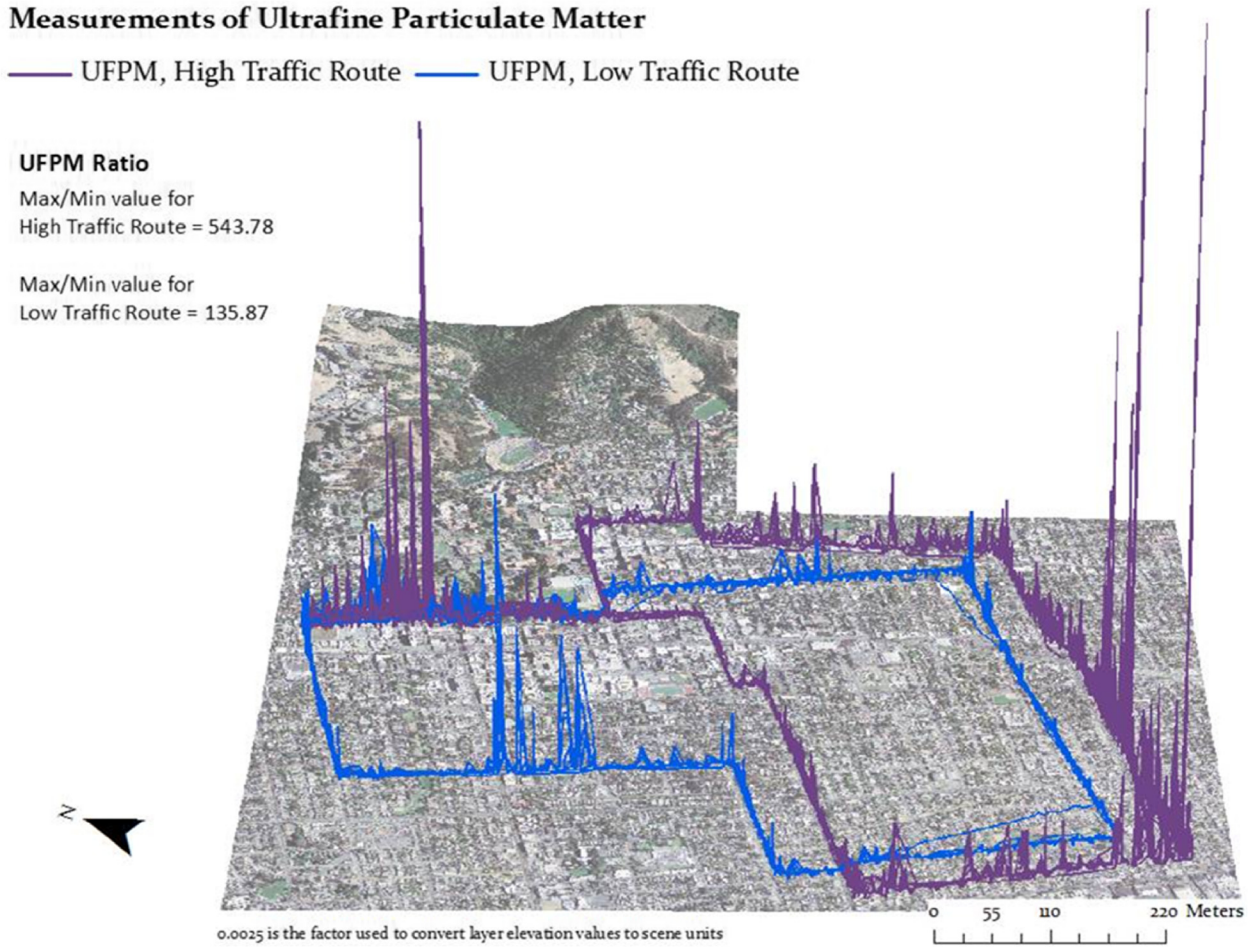
**UFPM concentrations on the low and high-traffic routes.** Low-traffic minimum: 2,771; low-traffic maximum: 376,495; High-traffic minimum: 1,900; high-traffic maximum: 1,033,188. Measurements are in particles per cubic centimeter.

We visualized spatial pollutant concentration trends by plotting the aggregated 50 m block data. Figure 3 shows where the average pollutant levels spiked. These spikes were generally in locations along each route where vehicle traffic was particularly high. On the low-traffic route, the largest spikes occurred where the residential roads of the route crossed main streets. Elevated pollutant levels were consistently measured on Oxford Street (shared by both routes); these could be due to construction on and near the road in addition to morning commute traffic heading toward the University. On the high-traffic route, the highest pollutant levels were seen on Ashby Avenue, designated as a major road and truck route by the City of Berkeley. To visualize the full range of UFPM exposure, we also plotted the high and low-traffic route measurements without the percent deviation from average calculation (Figure 4).

### Meteorology

**Figure 5:**
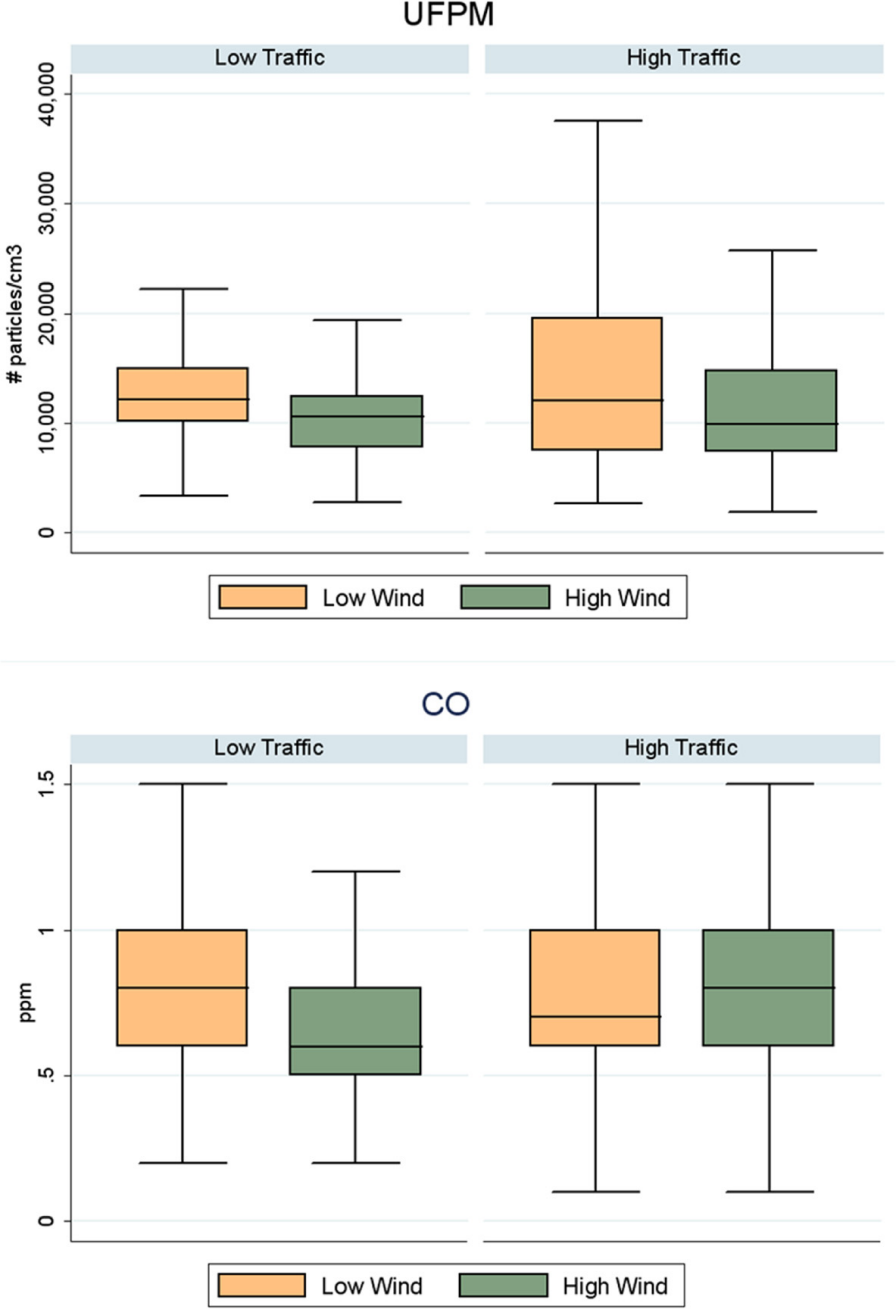
Box plots showing the difference between high and low-wind speed days for high and low-traffic averages.

We stratified the pollutant data by high-wind and low-wind days. We found that the average measurements on high-wind days and low-wind days were significantly different for UFPM, CO, and PM_2.5_. UFPM, CO, and BC averages were all higher on low-wind days. We further stratified the data by route (traffic level), and found that the high-traffic route on low-wind days had the highest average UFPM and CO measurements, while the low-traffic route on high-wind days had the lowest average concentrations of UFPM and CO. We have displayed the effect of wind speed on UFPM and CO concentrations independent of traffic volume in box plots (Figure 5).

### Health measurements

Baseline average forced vital capacity (FVC), forced expiratory volume in one second (FEV_1_), their ratio (FEV_1_/FVC =%FEV_1_), and forced expiratory flow rate between 25-75% of vital capacity (FEF_25-75%_) were calculated using the valid data. Post-ride and 4-hour follow-up measurements were also averaged and the changes from baseline were calculated for all four measures of lung function. Lung function data are displayed in Table 5. Average changes in lung function ranged from −0.1 liters (low-traffic post-ride FEF_25-75%_) to +0.24 liters (high-traffic 4-hour FEF_25-75%_), but all changes in lung function measurements were clinically insignificant, and none of the paired *t*-tests (by subject) for low-traffic and high-traffic lung function changes had significant p-values.

**Table 5:** Average measurements and changes in lung function

**Measurement**	**Low-traffic**	**High-traffic**
**FVC (liters)**		
Baseline ± SD	4.90 ± 0.71	5.01 ± 0.83
Post-ride (Δ from baseline)	4.88 (−0.02)	5.01 (0.00)
4-hour (Δ from baseline)	4.87 (−0.03)	4.96 (−0.05)
**FEV**_**1**_**(liters)**		
Baseline ± SD	3.91 ± 0.60	3.95 ± 0.62
Post-ride (Δ from baseline)	3.93 (0.02)	4.00 (0.05)
4-hour (Δ from baseline)	3.95 (0.04)	3.94 (−0.01)
**FEV**_**1**_**/FVC (liters)**		
Baseline ± SD	0.81 ± 0.07	0.79 ± 0.06
Post-ride (Δ from baseline)	0.81 (0.00)	0.80 (0.01)
4-hour (Δ from baseline)	0.81 (0.00)	0.81 (0.02)
**FEF**_**25-75%**_**(liters)**		
Baseline ± SD	3.87 ± 0.94	3.61 ± 0.91
Post-ride (Δ from baseline)	3.77 (−0.10)	3.78 (0.17)
4-hour (Δ from baseline)	3.94 (0.07)	3.85 (0.24)

## Discussion

### Air pollution measurements

We found significantly elevated concentrations of ultrafine particulate matter and carbon monoxide, and borderline significant differences for black carbon on the high-traffic route relative to the low-traffic route. When we spatially plotted the pollutant measurements using GPS coordinates, we also found that spikes in pollutant concentrations occurred mainly at intersections and busy streets or truck routes. This supports our hypothesis that traffic volume is directly related to outdoor pollutant concentrations and suggests that route choice can affect commuter exposure to pollutants associated with vehicle exhaust. These findings are consistent with previous European and Canadian studies comparing pollution exposure levels on routes of different traffic levels [9,10,22], and our measured exposure difference between high-traffic and low-traffic routes was comparable to these previous studies. For example, the mean ultrafine particular matter (in particles per cubic centimeter, also referred to as particle number) measurements in our study were: 14,311 (low-traffic) and 18,545 (high-traffic). Strak, et al. reported means of 27,813 (low) and 44,090 (high); Weichenthal, et al. 10,882 (low) and 19,747 (high); and McCreanor, et al. 18,300 (low) and 63,700 (high) [9,10,23]. The high to low traffic ratios are: 1.29 (this work), 1.62, 1.81, and 3.48, respectively. Zuurbier, et al. calculated 1.6-2.5 times higher particle number on their high-traffic route [24]. Our maximum value was 1,033,188 particles per cubic centimeter, indicating the potential for extraordinarily high exposures over 10-second intervals. Potential health effects of such spikes are unknown, but may merit attention in future research.

We also found that wind speed was associated with significant differences in average measured pollutant concentrations, suggesting that weather conditions also impact the pollution exposure levels of bicycle commuters. Time of day is another important factor – avoiding rush-hour periods significantly reduces cyclists’ pollution exposure [22].

### Health effects of pollutant exposure

Although we did not find significant changes in pulmonary function after cycling on either route, this is not surprising given that our subjects were healthy and screened to not have asthma. A previous study demonstrated reduced lung function and elevated levels of biomarkers of inflammation associated with increased pollutant concentrations in asthmatic subjects who walked along an inner city roadway with high-volume diesel-powered traffic [23], though these findings were not reproduced in a study with non-asthmatic study subjects bicycling on routes with different traffic levels [9]. In studies looking at other pulmonary and cardiovascular outcomes in healthy subjects, findings have been inconclusive. Zuurbier, et al. (2011) did not find consistent associations between commuting-related air pollution exposure and observed biomarkers including Clara cell protein 16, blood cell count, coagulation markers, and inflammation markers [24]. Similarly, in a comparison of cyclist exposure on a busy road versus a laboratory with filtered air, Jacobs, et al. (2010) did not observe a significant difference in Clara cell protein 16, exhaled nitric oxide (NO), plasma IL-6, platelet function, and total blood leukocytes; however a small increase in inflammatory blood cells was observed [25].

### Limitations

A major limitation of this study was variable wind speed and other meteorological conditions, which affected measured concentrations independent of road traffic volume. Equipment failure also reduced the number of viable pollutant measurements. Participants did not cycle to the study site, but pre-study exposure and potential exposure between the post-ride and 4-hour follow-up spirometry measurements were not otherwise controlled. In particular, we recognize that allowing participants to drive to the study site may have influenced their pre-exposure to vehicle-related air pollutants. Finally, the exposure time on the routes may not have been long enough to elicit measurable physiological changes.

## Conclusions

This study contributes to a small but growing body of literature that investigates the network of relationships among active transport, air pollutant exposure, and health. More research in this field is still needed to more conclusively determine the long-term health effects of bicycle commuting in variable traffic conditions. Our results indicate that choosing low-traffic routes can decrease the exposure of bicyclists to air pollutants, potentially reducing associated detrimental health effects. In particular, this study demonstrates that even simple infrastructure modifications, such as designating a particular network of streets as “bicycle boulevards,” have the potential to mitigate exposure to pollution. Implementing a system of bicycle boulevards does not require the construction of new streets or bike paths, nor does it necessitate the narrowing of existing lanes of traffic, which are often major barriers to implementing effective bicycling infrastructure. Lower pollutant exposures on bicycle boulevards could increase the number of cyclist commuters, especially in subpopulations more vulnerable to the health effects of air pollution, such as asthmatics. An increase in bike commuting could in turn decrease the risk of vehicle-bicycle collision, as studies have shown that higher numbers of cyclists raise awareness and lower the rate of accidents, i.e. the “safety in numbers” effect [26,27]. Lower pollutant exposures associated with low-traffic routes and bicycle boulevards may support future changes to the built environment (city, building, and street design) that allow for healthier and safer routes for active transportation.

## Abbreviations

GPS: Global positioning system; CO_2_: Carbon dioxide; PM_2.5_: Fine particulate matter (diameter ≤ 2.5 micrometers); UFPM: Ultrafine particulate matter (diameter <0.1 micrometers); NO_2_: Nitrogen dioxide; O_3_: Ozone; EPA: Environmental Protection Agency; VMT: Vehicle miles traveled; CF: Correction factor; BC: Black carbon; CPC: Condensation particle counter; ATS: American Thoracic Society; ERS: European Respiratory Society; FVC: Forced vital capacity; FEV_1_: Forced expiratory volume in 1 second; FEV_1_/FVC: Ratio of FVC over FEV_1_; FEF_25-75_: Forced expiratory flow rate between 25-75% of vital capacity.

## Competing interests

The authors declare that they have no competing interests.

## Authors’ contributions

SJ collected and analyzed data and drafted the manuscript; MJ conceived of the study, oversaw study design and data analysis, assisting with clinical data collection, and assisted with writing the manuscript; DW oversaw collection of exposure data and data analysis and assisted with writing the manuscript; AN assisted with study design, analysis of data, and writing the manuscript; CH collected data and assisted with writing the manuscript; LD collected data and performed GIS analysis; JL oversaw and assisted with GIS analysis; JB oversaw study design and collection and analysis of spirometry data and assisted with writing the manuscript. All authors read and approved the final manuscript.
